# The complete chloroplast genome of *Drymocallis saviczii* (Rosaceae: Potentilleae)

**DOI:** 10.1080/23802359.2020.1756966

**Published:** 2020-05-12

**Authors:** Ya-Kun Li, Khasbagan, Qin-Qin Li

**Affiliations:** College of Life Science and Technology, Inner Mongolia Normal University, Hohhot, Inner Mongolia, China

**Keywords:** Chloroplast genome, *Drymocallis saviczii*, Potentilleae, Rosaceae

## Abstract

The complete chloroplast genome of *Drymocallis saviczii* was reported in the present study. The chloroplast genome of *D. saviczii* was a circular DNA molecule with a size of 154,487 bp in length. The genome had a typical quadripartite structure composed of a pair of inverted repeats (IRa and IRb) of 25,991 bp separated by a large single-copy (LSC) region of 84,332 bp and a small single-copy (SSC) region of 18,173 bp. The genome encoded a set of 129 genes, comprising 84 protein-coding genes, 37 tRNA genes, and eight rRNA genes. Phylogenetic analysis demonstrated that *D. saviczii* was closer to *D. glandulosa* in current sampling.

*Drymocallis saviczii* (Schischk. et Kom.) Soják [synonym: *Potentilla saviczii* Schischk. et Kom.] belongs to the family Rosaceae Juss., subfamily Rosoideae (Juss.) Arn., tribe Potentilleae Sweet. This species is distributed in the eastern half of Siberia in the Russian Far East and adjacent parts of north-eastern China (Soják 2004, 2007, 2011, 2012). The complete chloroplast (cp) genome of *D. saviczii* was reported and characterized herein, which provides significant information for further studies on its taxonomy and population genetics.

Fresh leaves of *D. saviczii* were collected from Arxan, Nei Mongol, China. Voucher specimen (no. Li QQ 20170822002) was deposited in the herbarium of Inner Mongolia Normal University (NMTC). Total genomic DNA was extracted using the method of Doyle and Doyle (1987). Short-insert library (insert size, 300 bp) was prepared and then sequenced using the Illumina HiSeq platform in Novogene (Nanjing, China). A total of 36,860,588-bp raw reads were generated by Illumina paired-end sequencing after removing adapters. The raw reads were used to assemble the cp genome in NOVOPlasty (Dierckxsens et al. 2017), with ribulose-1, 5-bisphosphate carboxylase/oxygenase (*rbcL*) gene from *D. glandulosa* (GenBank accession no. KY420015) as the seed sequence. Chloroplast genome annotation was conducted using transferring annotations in Geneious Prime (Kearse et al. 2012), with the complete cp genome of *Farinopsis salesoviana* (Steph.) Chrtek et Soják (GenBank accession no. MT017928) as the reference. Where necessary, gene boundaries were manually corrected to match the start and stop codons and intron/exon boundaries. The annotated complete cp genome of *D. saviczii* was submitted to GenBank with the accession no. MT178809. The complete cp genome of *D. saviczii* was a circular DNA molecule with a size of 154,487 bp in length. The genome had a typical quadripartite structure, which composed of two copies of inverted repeats (IRa and IRb: 25,991 bp) separated by two regions: the large single-copy region (LSC: 84,332 bp) and the small single-copy region (SSC: 18,173 bp). The total GC content was 37.2%, and those of the LSC, SSC and each IR were 35.1%, 30.9%, and 42.8%, respectively. The cp genome encoded a set of 129 genes, comprising 84 protein-coding genes, 37 tRNA genes, and eight rRNA genes.

To examine the phylogenetic position of *D. saviczii*, the cp genome sequences of *D. saviczii* and 31 related species in Potentilleae plus four *Rosa* species were aligned by MAFFT version 7.450 (Katoh and Standley 2013) and trimmed properly using trimAL version 1.4 (Capella-Gutiérrez et al. 2009). The maximum likelihood (ML) tree was inferred using RAXML version 8 (Stamatakis 2014), with the combined rapid bootstrap analysis (1000 replicates) and search for best-scoring ML tree (the ‘-f a’ option). The GTRGAMMA model was used in the analysis. Phylogenetic tree demonstrated that *D. saviczii* was closer to *D. glandulosa* than to other species in current sampling (Figure 1).

**Figure 1.**
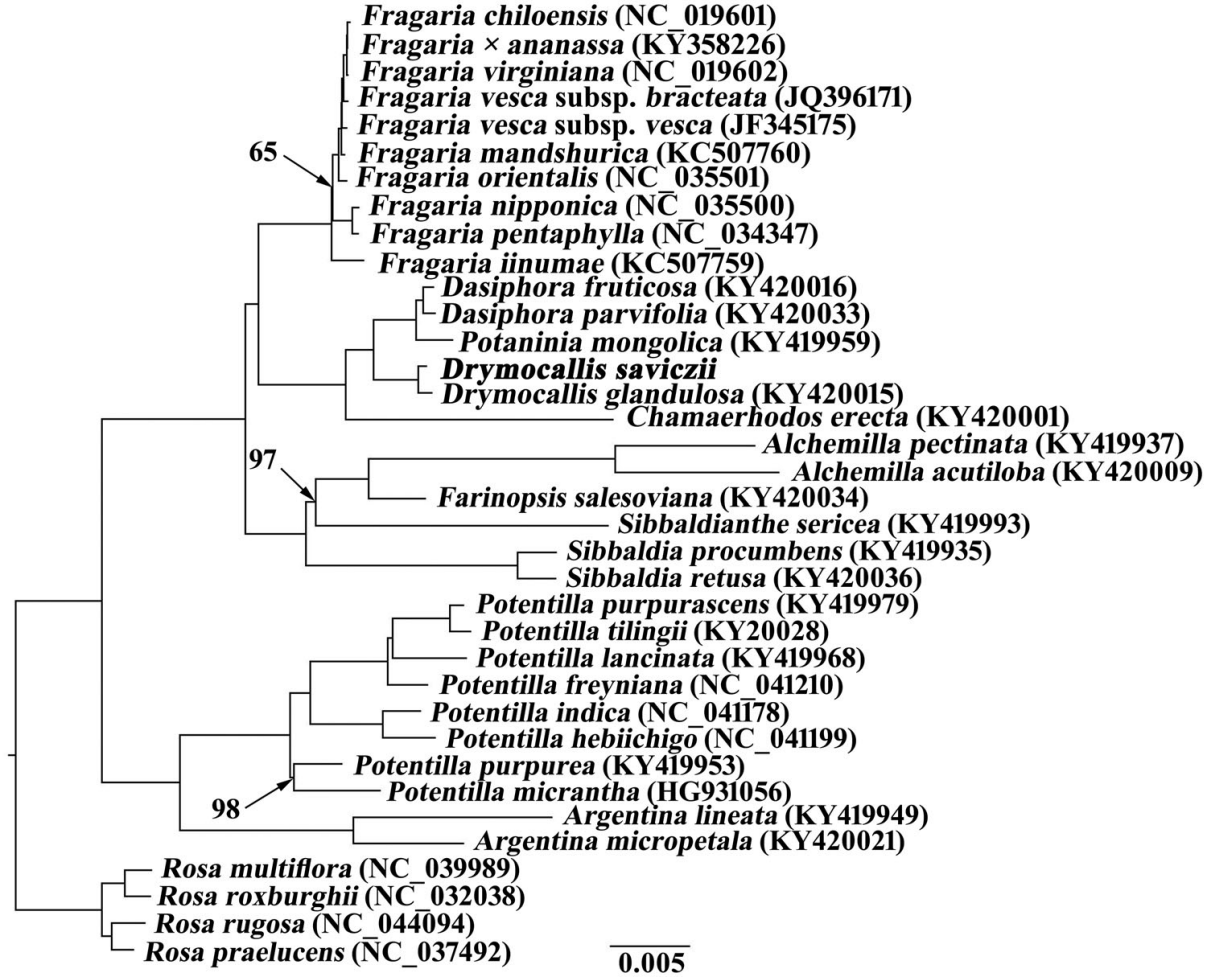
Maximum likelihood (ML) tree based on the cp genome sequences of *Drymocallis saviczii* and related species plus four Rosa species as outgroups. Values along branches correspond to ML bootstrap percentages (only values <100% are shown).

## Data Availability

The authors confirm that the data supporting the finding of this study are available within its supplementary material.

## References

[CIT0001] Capella‐Gutiérrez S, Silla‐Martínez JM, Gabaldón T, 2009. trimAl: a tool for automated alignment trimming in large‐scale phylogenetic analyses. Bioinformatics. 25(15):1972–1973. 19505945 10.1093/bioinformatics/btp348 PMC2712344

[CIT0002] Dierckxsens N, Mardulyn P, Smits G. 2017. NOVOPlasty: de novo assembly of organelle genomes from whole genome data. Nucleic Acids Res. 45(4):e18. 28204566 10.1093/nar/gkw955 PMC5389512

[CIT0003] Doyle JJ, Doyle JL. 1987. A rapid DNA isolation procedure for small amounts of fresh leaf tissue. Phytochem Bull. 19(1):11–15.

[CIT0004] Katoh K, Standley DM. 2013. MAFFT multiple sequence alignment software version 7: improvements in performance and usability. Mol Biol Evol. 30(4):772–780. 23329690 10.1093/molbev/mst010 PMC3603318

[CIT0005] Kearse M, Moir R, Wilson A, Stones‐Havas S, Cheung M, Sturrock S, Buxton S, Cooper A, Markowitz S, Duran C, et al. 2012. Geneious Basic: an integrated and extendable desktop software platform for the organization and analysis of sequence data. Bioinformatics. 28(12):1647–1649. 22543367 10.1093/bioinformatics/bts199 PMC3371832

[CIT0006] Soják J. 2004. *Potentilla* L. (Rosaceae) and related genera in the former USSR (identification key, checklist and figures). Notes on *Potentilla* XVI. Bot Jahrb Syst. 125(3):253–340.

[CIT0007] Soják J. 2007. *Potentilla* (Rosaceae) in China. Notes on *Potentilla* XIX. Harv Pap Bot. 12(2):285–324.

[CIT0008] Soják J. 2011. Synopsis of *Drymocallis* Fourr. ex Rydb. (Rosaceae–Potentilleae) in the old world. Ann Naturhist Mus Wien, B. 112:319–328.

[CIT0009] Soják J. 2012. *Potentilla* L. (Rosaceae) and related genera in Asia (excluding the former USSR), Africa and New Guinea. Notes on *Potentilla* XXVIII.Plant Div Evol. 130(1-2):7–157.

[CIT0010] Stamatakis A. 2014. RAxML version 8: a tool for phylogenetic analysis and post-analysis of large phylogenies. Bioinformatics. 30(9):1312–1313. 24451623 10.1093/bioinformatics/btu033 PMC3998144

